# Supplementary figures and images for: In-water observations highlight the effects of provisioning on whale shark behaviour at the world's largest whale shark tourism destination
Source: R Soc Open Sci. 2020 Dec 16;7(12):200392. doi: 10.1098/rsos.200392 (PMC7813242; doi:10.1098/rsos.200392)

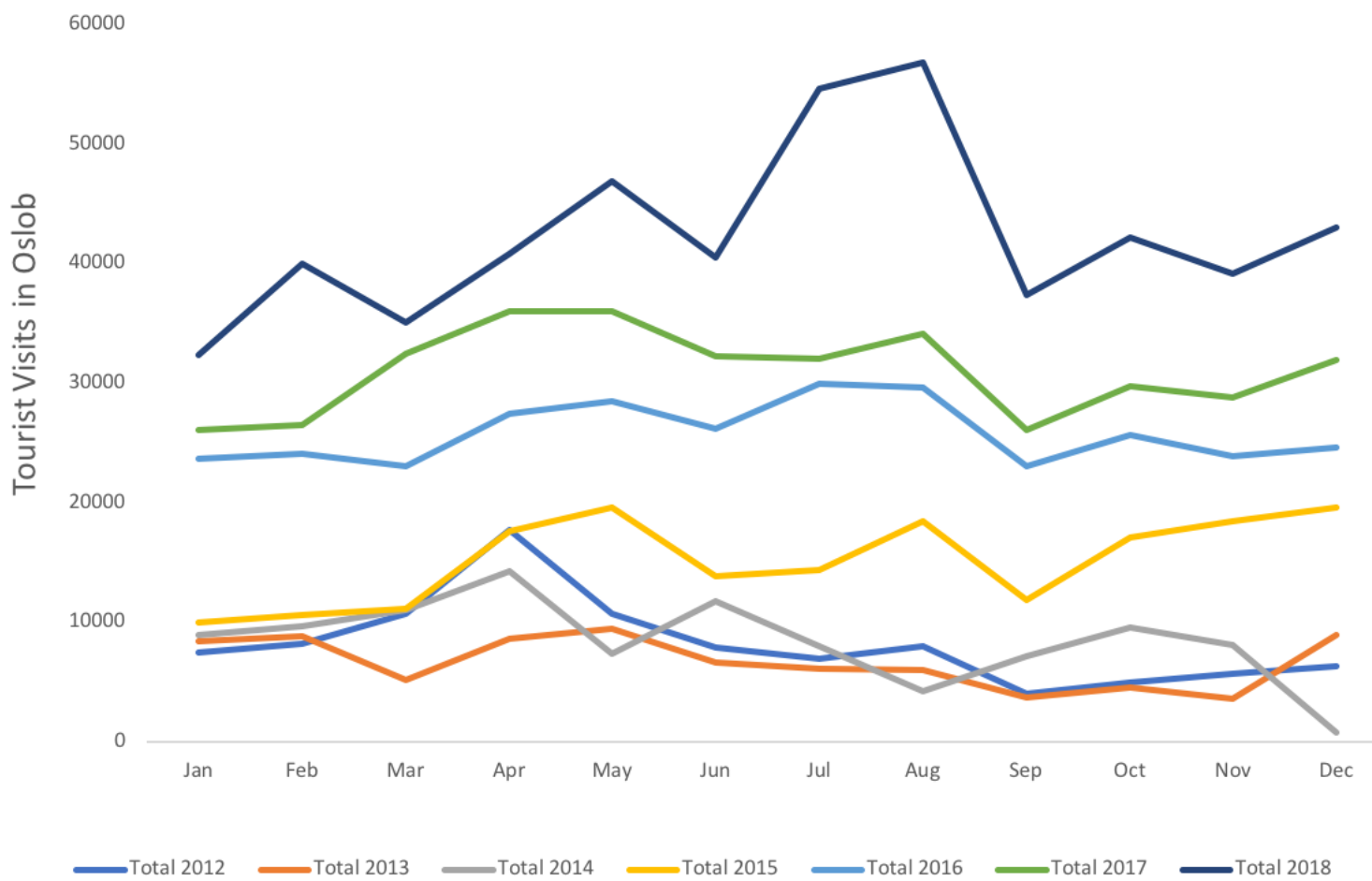

Supplement: Figure S1 [file rsos200392supp1.pdf]

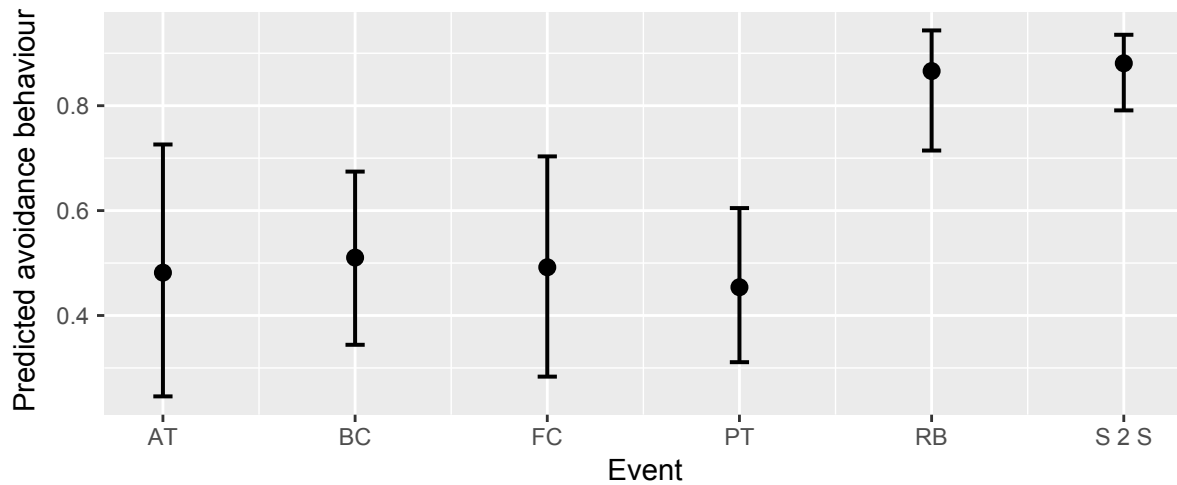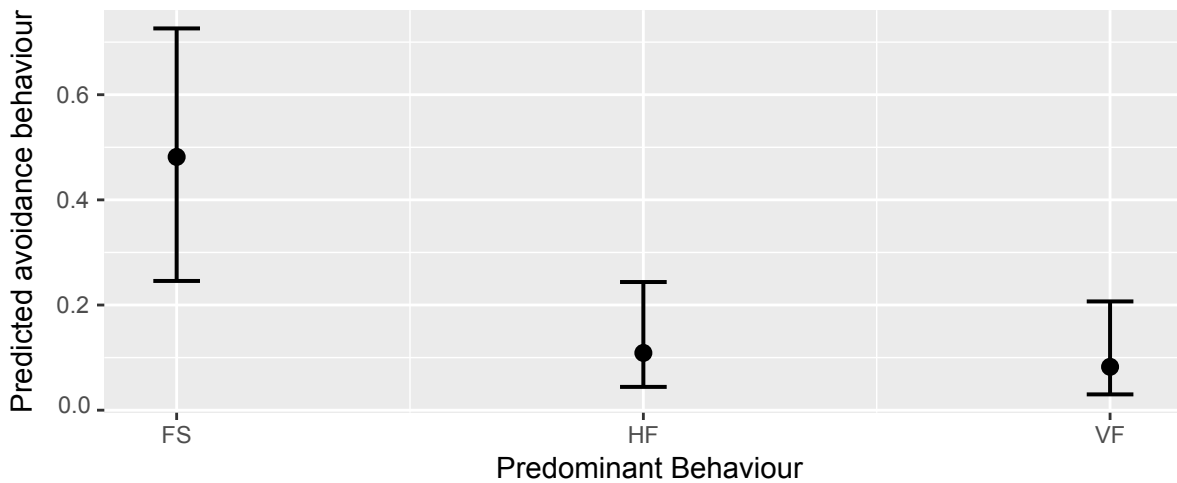

Supplement: Figure S2 [file rsos200392supp2.pdf]
